# Whole-genome sequencing of Lassa virus from dry blood spots: a comparative evaluation

**DOI:** 10.1186/s40249-025-01362-0

**Published:** 2025-10-13

**Authors:** Umaru Bangura, Christopher Davis, Andreas Dahl, Sylvia Klemroth, Emma Thomson, N.’Faly Magassouba, Elisabeth Fichet-Calvet

**Affiliations:** 1 Zoonoses Control Group, Bernhard Nocht Institute for Tropical Medicine, 20359 Hamburg, Germany; 2 School of Public Health, College of Medical Sciences, Njala University, Bo, Sierra Leone; 3 Centre for Virus Research, University of Glasgow, Glasgow, G61 1QH UK; 4 Dresden-Concept Genome Center, Dresden, Germany; 5 Laboratoire des Fièvres Hémorragiques en Guinée, Conakry, Guinea

**Keywords:** Lassa virus, *Mastomys natalensis*, Dry blood, Whole blood, Genomic sequencing, Guinea, West Africa

## Abstract

**Background:**

Whole blood samples are often used to generate whole genome sequences, which provide valuable insights into the genetic make-up of viruses. However, the collection and management present significant challenges, particularly in remote and resource-limited communities, where maintaining a cold chain is often difficult and costly. The use of dry blood spots (DBS) is gradually increasing to overcome these logistical barriers with reduced biosafety constraints. We propose an alternative approach using native DBS Lassa virus (LASV)-positive samples as a substitute for whole blood.

**Findings:**

Next-generation sequencing (NGS) was performed on RNA extracted from whole blood and DBS samples using Illumina technology. RNA concentration, cycle threshold (Ct) values and sequence read counts were statistically compared. A total of 78 samples from 39 LASV-positive *Mastomys atalensis* were analysed. Whole blood had significantly higher mean RNA concentration (26.5 ± 1.9) than DBS (3.4 ± 0.3), *P* < 0.05. Mean Ct values in whole blood were significantly lower than in DBS (*P* = 0.0001). Log mean sequence reads and NGS coverage for both S and L segments were significantly higher in whole blood (*P* = 0.0001). RNA concentration showed no association with sequence coverage (*P* = 0.382), while Ct values showed a strong association (*P* = 0.0001).

**Conclusions:**

Our study demonstrates that DBS is a viable alternative for whole genome sequencing of LASV, although whole blood samples consistently outperform DBS in terms of RNA concentration, Ct values and NGS coverage.

**Graphical Abstract:**

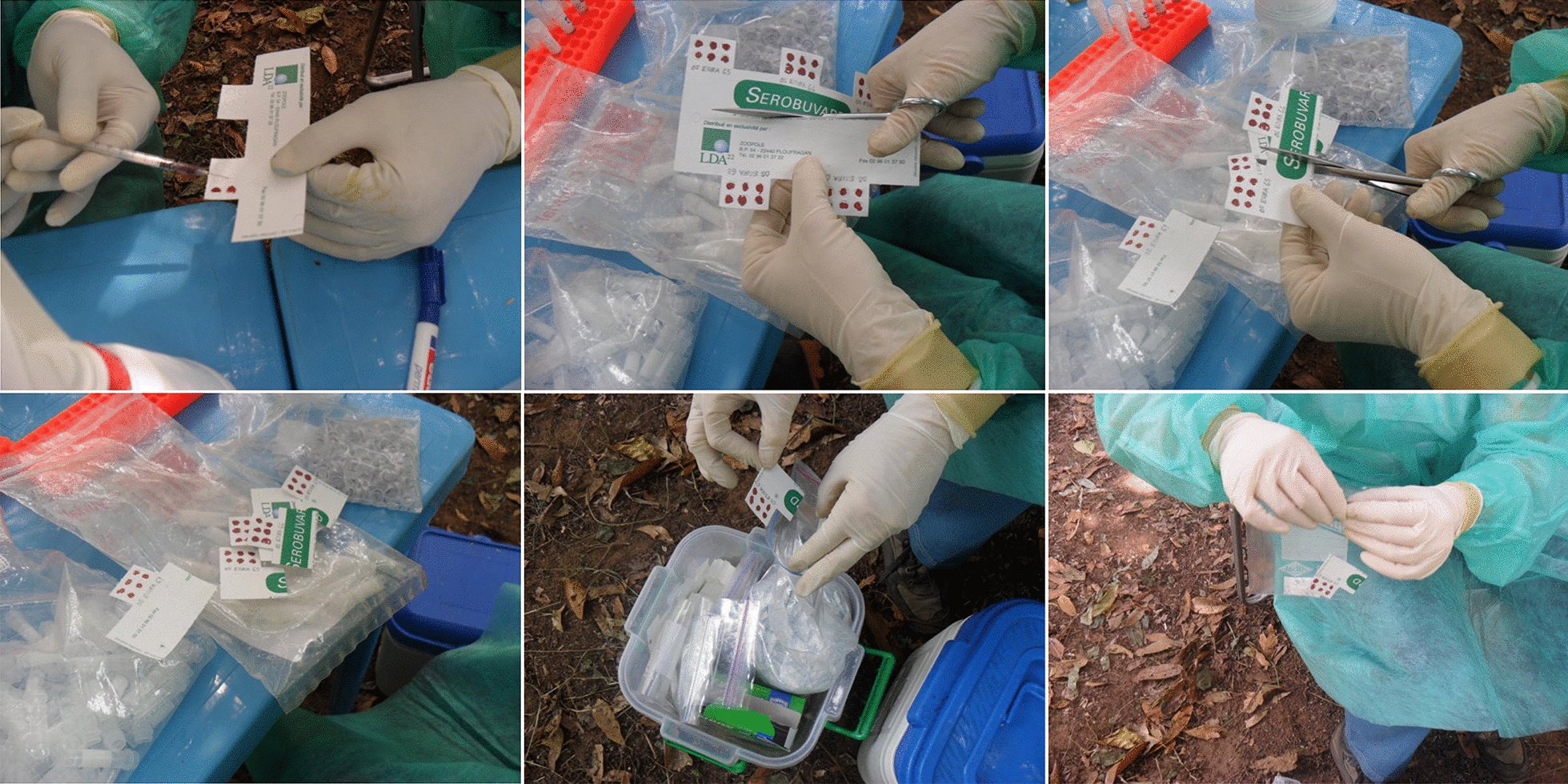

**Supplementary Information:**

The online version contains supplementary material available at 10.1186/s40249-025-01362-0.

## Background

High-throughput sequencing, particularly RNA sequencing, has transformed genomic research by enabling high-resolution transcriptome analysis. The quantity and quality of RNA are critical for accurate assessment of gene expression, regulation, and function. High RNA yields permit deeper transcriptome exploration, while low yields risk incomplete transcript representation and reduced sensitivity for detecting low-abundance transcripts [1]. RNA integrity, purity, and absence of contaminants are equally vital for reliable sequencing outcomes.

Cycle threshold (Ct) values from real-time reverse transcription polymerase chain reaction (RT-PCR) are frequently used to estimate viral RNA abundance. Lower Ct values correspond to higher viral loads; higher values indicate lower loads. However, degradation from enzymatic activity, environmental exposure, or mishandling can distort Ct measurements and impair sequencing by introducing bias and reducing genome coverage [2, 3].

Whole blood is a common source for viral genome sequencing, which informs variant characterization, virulence determinants, and transmission pathways [4–6]. In remote and resource-limited settings, however, maintaining a cold chain for whole blood is costly and logistically challenging. Dried blood spots (DBS) provide a practical alternative. Introduced in the 1960s for neonatal screening, DBS involves spotting small volumes of blood onto filter paper, air-drying, and sealing to prevent contamination [7]. Their use has expanded to molecular analysis, epidemiology, drug monitoring, proteomics, metabolomics, and pharmacokinetics [8–13].

DBS collection is inexpensive, stable at ambient temperature, and safer to handle than liquid blood. It requires no specialized equipment such as centrifuges or refrigeration, making it especially valuable for large-scale studies in remote regions [14–17].

Traditionally, complete viral genomes were obtained from cell cultures, which enrich viral RNA and minimize host contamination. Although effective, this requires lengthy protocols in biosafety level 4 (BSL-4) facilities for highly pathogenic agents such as Lassa virus (LASV). LASV, a member of the Arenaviridae family, causes Lassa fever (LF), endemic in parts of West Africa [18] with annual cases in Guinea, Liberia, Sierra Leone, and Nigeria [19–22]. It is estimated to infect ~ 897,700 people and cause ~ 18,000 deaths annually [23]. The primary reservoir, the Natal multimammate mouse (*Mastomys natalensis*), is widespread in West Africa and often inhabits homes during the dry season [24–28]. Human infection occurs through contact with rodent excreta, preparation of infected rodents as food, or inhalation of contaminated dust [29–33].

To circumvent BSL-4 constraints, we propose using native LASV-positive DBS samples in place of whole blood. This simplifies sample handling and reduces biosafety requirements [34]. We validated the approach by performing NGS on RNA from both whole blood and DBS. While our validation used *M. natalensis* samples, comparable outcomes are expected for human LASV DBS. Supporting this, serum from a fatal LF case in Guinea (2019) was dried on filter paper and later sequenced via NGS in Hamburg, Germany [35]. Similarly, three human DBS samples were successfully used to sequence Hepatitis C Virus subtype 2a at the University of Glasgow [36].

## Methods

### Rodent sampling and blood collection

Rodents were sampled in nine villages in the Faranah region of Guinea, each with a population of 600–1,000 inhabitants and located 30 km away from the nearest urban area. Sampling was carried out between 2013 and 2014. Live rodent trapping was conducted inside houses using Sherman traps (H.B. Sherman Trap Co., Tallahassee, FL, USA) for three consecutive nights. Traps were baited with a mixture of peanuts, dried fish, and wheat flour, set in the evening and collected the following morning [28]. Captured rodents were handled according to established guidelines [37, 38]. They were first anaesthetised with isoflurane, after which blood was collected via cardiac puncture. Blood samples were processed in two ways: some were dripped onto filter paper (SEROBUVARD-LABOCEA, Ploufragan, France) and air dried to produce DBS, while the remaining blood was stored in collection tubes. DBS samples were stored at −20 °C while whole blood samples were stored at −80 °C.

### LASV testing

RNA was extracted from whole blood and DBS samples using QIAamp Viral RNA kit (QIAGEN, Hilden, Germany), with carrier RNA replaced by linear acrylamide (ThermoFisher Scientific, Waltham, MA, USA). For DBS, a single spot per animal was added directly to Amplification Viral Lysis (AVL) buffer without prior elution. RNA concentrations (1 µl of each extract) were measured in ng/ml using a Qubit 4 fluorometer with the Qubit RNA HS (High Sensitivity) Assay Kit (ThermoFisher Scientific, Waltham, MA, USA) and assessed for quality using the Bioanalyzer RNA Kits and Reagents (Agilent Technologies, Santa Clara, CA, USA). RNA extracts were tested for the presence of LASV using the One-Step RT-PCR kit (Qiagen, Hilden, Germany) with Lassa-specific assays, targeting both the S segment [39] and the L segment [40] of the LASV genome. Ct values were obtained by real-time RT-PCR using the RealStar Lassa Virus RT-PCR Kit 2.0 (altona Diagnostics, Hamburg, Germany) on a Rotor-Gene Q (QIAGEN, Hilden, Germany), targeting both the GPC and L genes according to the manufacturer’s instructions.

### Sequencing of LASV strains

Sanger sequencing was performed by a private service (LGC, Berlin, Germany) to serve as a control for the NGS using amplicons obtained via conventional PCR. For the NGS, matched RNA extracts from whole blood and dried blood spots were delivered to the MRC-University of Glasgow Centre for Virus Research (CVR) in 2019 (*n* = 7, Batch 1) and to the Dresden-concept Genome Center (DcGC) in 2023 (*n* = 32, Batch 2).

CVR procedure: First-strand cDNA was synthesised using the Superscript III (ThermoFisher Scientific, Waltham, MA, USA). A 10 µl sample was combined with 1 µl of 50 ng/µl random hexamers and 10 mmol/L dNTP mix, incubated at 65 °C for 5 min, and cooled on ice for 1 min. The following were added: 4 µl of 5 × RT buffer, 2 µl of RT enzyme, 1 µl of RNaseOUT (40 U/µl), and 1 µl of 0.1 mol/L DTT. The reaction mixture was incubated at 25 °C for 10 min, 55 °C for 60 min and 70 °C for 15 min to complete the first-strand cDNA synthesis. Second-strand cDNA was performed using the NEBNext kit (New England BioLabs, Hitchin, UK) by adding 8 µl of 10 × second-strand synthesis buffer, 4 µl of second-strand synthesis enzyme mix, and 48 µl of nuclease-free water to the first-strand cDNA template and incubated at 16 °C for 2.5 h. Illumina NGS libraries were prepared using the KAPA LTP Library Preparation Kit (KAPA Biosystems, Wilmington, MA, USA). Starting with fragmented double-stranded cDNA, end repair and A-tailing procedures were performed. The cDNA was purified using 0.9 × AMPure XP (Beckman Coulter, Brea, CA, USA) magnetic beads (Beckman Coulter), and the DNA concentration was measured using a Qubit 2.0 fluorometer (ThermoFisher Scientific, Waltham, MA, USA), according to the manufacturer's instructions. Adapter ligation was performed, and libraries were amplified using KAPA HiFi HotStart ReadMix and a primer mix (KAPA Biosystems, Wilmington, MA, USA). Purification of the amplified libraries was carried out using AMPure XP magnetic beads at a 0.9∶1 beads-to-sample ratio. Samples were incubated for 5 min at room temperature, and the libraries were eluted with a final volume of 13 µl. Libraries were quantified using Qubit and Tape station analysis (Agilent Technologies, Santa Clara, CA, USA). Sequencing was performed on the Illumina MiSeq platform (Illumina, San Diego, CA, USA) at the CVR.

DcGC procedure: Second-stand cDNA, library preparation and fragment analysis were performed similarly to the CVR. In addition, DcGC performed the ribosomal RNA depletion from mouse/rat and bacteria. All but a maximum of 250 ng DNase-treated RNA was subjected to ribosomal depletion with a combined probe set against host and microbial rRNA using the NEBNext rRNA Depletion Kit v2 (human/mouse/rat; New England Biolabs, E7400X) and the NEBNext rRNA Depletion Kit (Bacteria, New England Biolabs, E7850X). Sequencing was performed on the Illumina NovaSeq 6000 using the NEBNext Ultra II RNA Library Prep Kit (New England BioLabs).

### Bioinformatics

Fastq files generated from the sequencing runs at CVR and DcGC were processed using de novo assembly, followed by reference mapping to whole-genome LASV using Tanoti software (http://www.bioinformatics.cvr.ac.uk/tanoti.php) [41, 42]. We assessed the quality of the NGS reads by comparing them to the Sanger-generated sequences through assembly and alignment using the MacVector v18.6.4 software (Mac Vector, Inc., Apex, NC, USA).

### Statistical analysis

Comparative analysis of the RNA concentration, Ct values and sequences read counts for both sample types (DBS and whole blood) were performed using Stata/IC 15.1 software (StataCorp 4905 Lakeway Dr, College Station, TX, USA), with a 5% significance level. A paired t-test was used to determine whether there was significant difference between the means of the RNA concentrations, Ct values, and sequence read counts for the DBS and WB samples. Linear regression was used to assess the influence of RNA concentration, Ct values and sequence read counts on sequence coverage. The model used sequence coverage (percentage) as the dependent variable, and RNA concentration, Ct values, and sequence read counts as independent variables.

## Results

**Fig. 1:**
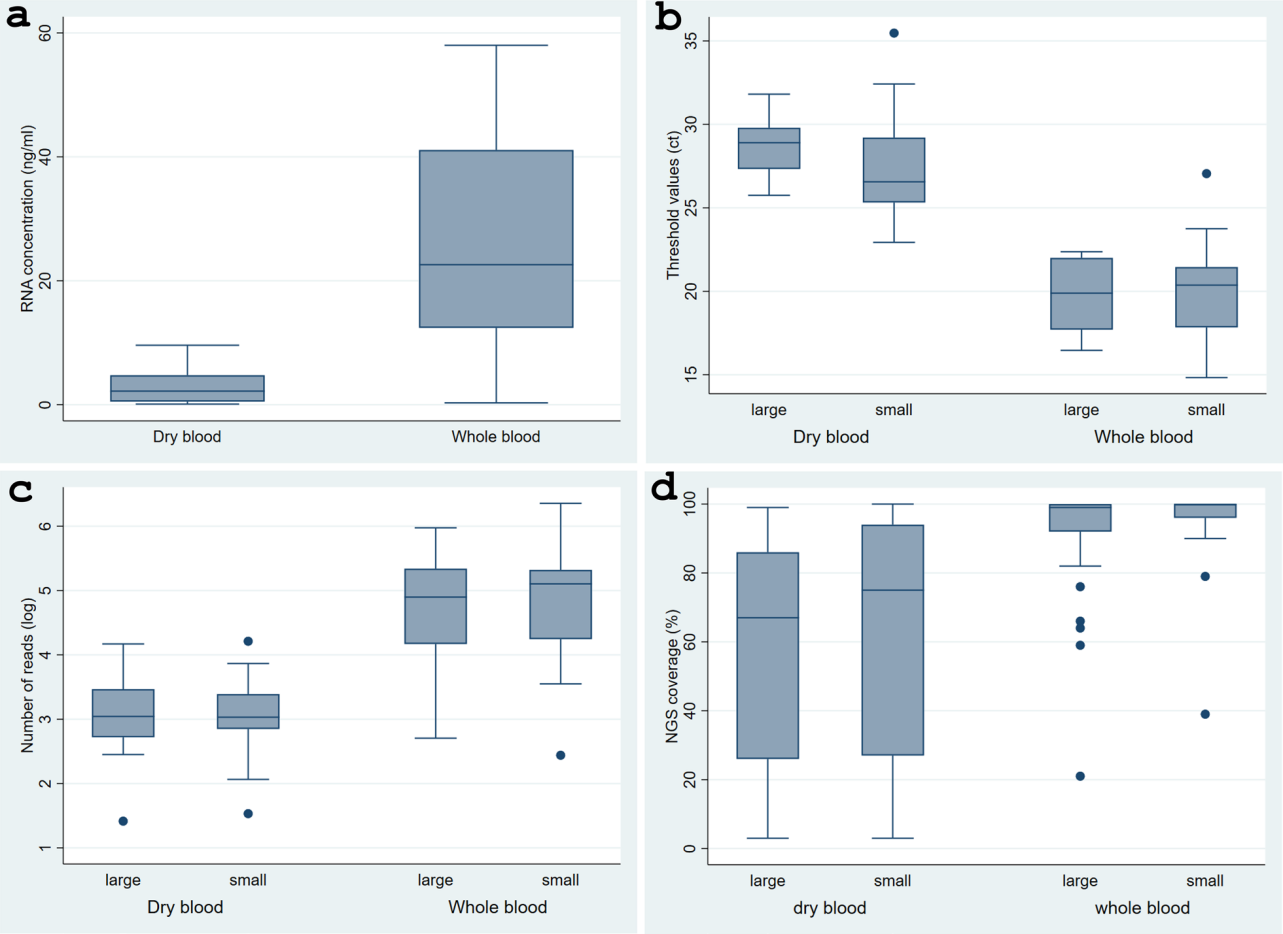
Comparison of dry blood and whole blood for several parameters: **a** RNA concentration (in ng/ml), **b** Ct value, **c** number of reads (log-transformed) and **d** NGS coverage (in %). Coverage was estimated by comparison with strain Bantou 366 accession numbers GU 979513 and GU 830839 for large and small segments, respectively

A total of 78 samples from 39 LASV-positive *M. natalensis* were analysed to compare RNA concentrations between whole blood (*n* = 39) and DBS (*n* = 39). RNA concentrations in whole blood samples ranged from 4.4 to 53 ng/ml, while DBS samples had concentrations ranging from 0.1 to 5.5 ng/ml. A paired t-test revealed that whole blood samples had a significantly higher mean RNA concentration (26.5 ± 1.9) compared to DBS samples (3.4 ± 0.3, 95% *CI:* 2.2–3.4), *t* (77) = 12.7, *P* < 0.05 (Fig. 1a, and additional file 1).

### Ct values

Positive controls included in the test kits had Ct values ranging from 30 to 32. For whole blood, the mean Ct value was 19.8 ± 0.4 (SD = 2.6, 95% *CI:* 19.0–20.7) for the S segment and 19.6 ± 0.7 (SD = 2.2, 95% *CI:* 17.9–21.3) for the L segment. In comparison, DBS samples exhibited higher mean Ct values of 27.1 ± 0.4 (SD = 2.8, 95% *CI:* 26.2–28) for the S segment and 28.6 ± 0.8 (SD = 2, 95% *CI:* 27.0–30.1) for the L segments (Fig. 1b and 2b in supplement). Statistical analysis confirmed that mean Ct values for both segments were significantly higher in DBS samples than in whole blood (*t* = 12.9, *P* = 0.0001 for S segment; t = 8.9, *P* = 0.0001 for L segment).

### Sequence reads

The analysis compared sequence reads from both the S and L segments of the LASV genome between DBS and whole blood samples (Fig. 1c and additional file 1). For the S segment, sequence reads in DBS ranged from 34 to 16,275 (mean = 2302), whereas reads in whole blood ranged from 275 to 2,265,547 (mean = 26,7436). For the L segment, reads ranged from 26 to 14,788 (mean = 2210) in the DBS and from 507 to 943,068 (mean = 178,043) in whole blood. Due to the wide range of sequence reads, values were log transformed (Log_10_) for analysis. The transformed mean log sequence reads for whole blood were 4.8 (95% *CI*: 4.7–5.1) and 4.7 (95% *CI*: 4.4–5.0) for the S and L segments, respectively. For DBS, the transformed mean was 3.0 (95%* CI*: 2.8–3.3) for both segments. Statistically, the log mean sequence reads were significantly higher in whole blood than compared to DBS for both S and L segments (*t* = −8.45, *P* = 0.0001 for S segment; *t* = −6.3, *P* = 0.0001 for L segment, Fig. 1c).

### NGS coverage

The analysis included both the S and L segments of the LASV genome from DBS and whole blood samples (Fig. 1d). The mean NGS coverage from the whole blood was 96.3% (range 39–100, *n* = 39, 95% *CI:* 93.0–99.6) for the S segment and 92% (range 21–100, *n* = 39, 95% *CI:* 87–97) for the L segment. In contrast, DBS samples showed lower mean NGS coverage, with 63% (range: 3–100, *n* = 39, 95% *CI:* 52.2–73.8] for the S segment and 56% (range: 3–99, *n* = 39, 95% *CI:* 46.1–66.6) for the L segment. Statistical analysis confirmed that NGS coverage was significantly higher in the whole blood compared to DBS for both the S and L segments (*t *= 5.69, *P* = 0.0001 for S segment; *t *= 6.1,* P* = 0.0001 for L segment).

### Relationship between RNA concentration, Ct values, sequence reads, and coverage

A linear regression analysis was performed to examine the relationship between RNA concentration, Ct values, and sequence reads with sequence coverage of the LASV genome. The analysis revealed that RNA concentration was not statistically associated with sequence coverage (*P* = 0.382). In contrast, Ct values showed a highly significant association with sequence coverage (*P* = 0.0001), indicating that lower Ct values (reflecting higher viral loads) are strongly correlated with increased sequence coverage. Sequence reads showed weak statistical significance for sequence coverage (*P* = 0.075), suggesting that while sequence reads may contribute to coverage, their effect is less consistent compared to Ct values.

### Duration of sample storage

The duration of sample storage compares the mean Ct and coverage in batch 1 (5.5 years) with batch 2 (9.5 years) (Table 1). The mean Ct for samples stored for 5.5 years was 23.7 (95% *CI*: 21.3–25.2), while the mean Ct was 23.7 (95% *CI*: 22.5–24.8) for samples stored for up to 9.5 years. There was no significant difference between the two means (*P* = 0.7). For sequence coverage, the mean values for the 5.5 and 9.5 years of storage were 82.5% (95%* CI*: 72.4–92.7) and 75.2% (95% *CI*: 69.6–80.7), respectively. This difference was not statistically significant (*P* = 0.2).

**Table 1:** NGS coverage and Ct values by type of biopsy (dry blood spot and whole blood), date of collection, date of extraction, date of sequencing, storage duration and temperature

Biopsy	*n*	Date of collection	Date of extraction	Date of sequencing	Storage duration	Temperature	NGS coverage
DBS	7	Nov–Dec 2013	June 2019	July 2019	5.5 years	−20 °C	75–99
WB						−80 °C	88–100
DBS	32	Nov 2013–April 2014	June 2023	Sep–Oct 2023	9.5 years	−20 °C	0–100
WB			Dec 2023	May 2024	10 years	−80 °C	39–100

NGS coverage in % includes both S and L segments

## Discussion

This study highlights the different effects of RNA concentration, Ct values, and sequence reads on sequence coverage in LASV sequencing. Of these variables, Ct values demonstrated the strongest correlation with sequence coverage, whereas RNA concentration showed no significant association, and sequence reads showed only a weak correlation. The lack of association between RNA concentration and sequence coverage highlights that RNA quantity alone is insufficient to predict sequencing success. This finding is consistent with previous research indicating that RNA quality and degradation have a greater impact on sequencing outcomes than raw RNA content [43].

In contrast, Ct values showed a highly significant association with sequence coverage. Our study demonstrated that Ct values strongly influence viral genome recovery, with the lowest Ct values achieving 100% recovery. Ct values reflect amplification efficiency during quantitative PCR, with lower values indicating higher viral loads and better RNA quality templates. This finding highlights the critical importance of high RNA quality with sufficient viral load for efficient genome sequencing. These results are consistent with the findings of Aitken et al. [44], who reported that Ct values are a strong predictor of sequencing success, particularly in viral genomics.

Although sequence reads showed low statistical significance, they still had a limited but significant influence on sequence coverage. Sequence reads are fundamental for genome assembly and coverage; however, variability in the sequencing process, such as read length, error rates, and adapter contamination, can reduce their overall contribution to consistent coverage [45]. The weaker association observed in this study suggests that although higher read counts can aid sequencing, their impact remains secondary to the quality of the initial RNA template, as reflected in Ct values.

Our results show that although DBS can be used to recover the complete Lassa virus genome, they are less efficient than whole blood. Several factors may account for this difference, including processing conditions, storage duration, and blood volume. In our study, DBS samples stored at −20 °C, were subjected to multiple freeze-thaw cycles during storage and handling. These cycles are likely to have contributed to the observed low viral RNA concentrations, as they are known to cause substantial RNA degradation. Freeze-thaw cycles can activate RNases and lead to fragmentation of the RNA molecules due to temperature fluctuations, which critically affects RNA integrity and yield, particularly in dried biological samples such as DBS [46, 47]. In addition, oxidative processes during DBS storage on filter paper can further accelerate RNA degradation [45]. The higher Ct values observed in DBS compared to whole blood underscore this susceptibility to viral RNA depletion and reduced RNA quality.

Whole genome sequencing was performed on our samples after 5.5 and 9.6 years of storage. The four years storage difference between the two batches shows no statistically significant effect in both Ct values and sequence coverage. This could be attributed to the limited freeze thaw cycle, as numerous molecular and serological tests were performed in the early years after collection. These results confirm that samples can remain stable when stored at frozen temperatures for long periods.

In this study, 140 µl of whole blood was used for the analysis, whereas only 10–20 µl of blood was used in DBS. This significant difference in blood volume is likely to limit the extraction capacity of viral RNA from DBS, thereby reducing the sensitivity of downstream molecular assays [7]. Larger sample volumes inherently provide more viral RNA input for extraction, increasing the likelihood of obtaining high-quality viral RNA for amplification and sequencing.

Despite its logistical advantages, such as ease of collection, storage, and transport, the limitations of DBS in viral RNA yield require optimisation strategies for wider application in molecular studies. Future research should focus on improving RNA stability during the drying and storage phases, including minimising freeze-thaw cycles by aliquoting, preserving samples at −80 °C, and optimising extraction protocols to maximise RNA recovery [48].

In conclusion, our findings underscore the critical role of Ct values in determining sequence coverage in viral genome sequencing, particularly for RNA viruses such as the Lassa virus. These results highlight the importance of optimised sample preparation protocols that prioritize viral RNA integrity and quality over raw concentration, as lower Ct values are strongly associated with greater sequencing success. In addition, our study demonstrates that DBS is a viable alternative for whole genome sequencing of LASV, although whole blood samples consistently outperform DBS in terms of RNA concentration, Ct values, and NGS coverage. Despite its limitations, DBS remains a valuable option in situations where sample collection conditions are challenging, such as fieldwork in remote areas with limited resources. Its logistical advantages, including ease of collection, storage, and reduced reliance on cold chain infrastructure, make DBS a practical solution for expanding access to viral genome sequencing in resource-limited settings.

## Supplementary Information

Supplementary material 1.

Supplementary material 2.

## Data Availability

All data generated or analysed during this study are included in this published article and its supplementary information files (additional files 1 and 2).

## Abbreviations

AVL: Amplification viral lysis
Ct: Cycle threshold
CVR: Centre for Virus Research
DBS: Dried blood spot
DcGC: Dresden-concept Genome Center
DNA: DeoxyriboNucleic acid
LASV: Lassa virus
NGS: Next generation sequencing
RNA: RiboNucleic acid
RT-PCR: Reverse transcription polymerase chain reaction
