# Supplementary material for: Whole-genome sequencing of Lassa virus from dry blood spots: a comparative evaluation
Source: Infect Dis Poverty. 2025 Oct 13;14:102. doi: 10.1186/s40249-025-01362-0 (PMC12516887; doi:10.1186/s40249-025-01362-0)
Supplement: Supplementary file 1 — Supplementary material 1. [file 40249_2025_1362_MOESM1_ESM.docx]

**Additional file 1**

**Whole-genome sequencing of Lassa virus from dry blood spots: a comparative evaluation**

Umaru Bangura^1,2^*, Christopher Davis^3^, Andreas Dahl^4^, Sylvia Klemroth^4^, Emma Thomson^3^, N’Faly Magassouba^5^ and Elisabeth Fichet-Calvet^1^*

**Figure S1**

**Figure S2**

**Figure S3**

**Figures S1-S3:** Next-generation sequencing coverage (in %) of the LASV genome obtained from whole blood and dry blood spots categorised by RNA concentration (Figure S1), Ct value (Figure S2), and the number of reads (Figure S3), for both S and L segments. The sequences were derived from *Mastomys naalensis* trapped in seven villages in Upper Guinea. The reference used to estimate the coverage was Bantou 366 (GU830839 and GU979513).
